# Indomethacin-Induced Hypertensive Crisis

**DOI:** 10.7759/cureus.18043

**Published:** 2021-09-17

**Authors:** Bibek Dhungana, Sajog Kansakar, Palak Paudel, Bibek Kc, Ashish Guragain

**Affiliations:** 1 Internal Medicine, KIST Medical College, Lalitpur, NPL; 2 Internal Medicine, Manipal College of Medical Sciences, Pokhara, NPL; 3 Gynaecology, Bhaktapur Cancer Hospital, Kathmandu, NPL

**Keywords:** stroke, prostaglandins, blood pressure, hypertension, indomethacin, nsaid

## Abstract

Non-steroidal anti-inflammatory drugs (NSAIDs) are one of the most commonly prescribed drugs to treat pain, and are easily available over the counter in lower dosages. NSAID use is associated with various side effects and elevated blood pressure is one of them. NSAIDs vary considerably in their effect on blood pressure with indomethacin being one of the NSAIDs associated with a significant increase in blood pressure. We present a case of a 58-year-old woman who developed a hypertensive crisis after a single dose of prescription indomethacin.

## Introduction

Non-steroidal anti-inflammatory drugs (NSAIDs) are among the most commonly used medications globally due to their easy availability, and effectiveness in resolving acute ailments like pain and fever, with a reported 29 million adults in the USA who are regular users of NSAIDs [1]. In light of their widespread use, the adverse effects of NSAIDs have continued to raise concerns and include gastrointestinal ulceration, renal toxicity, stroke, myocardial infarction, heart failure, bronchospasm, and neutropenia [2]. It has also been shown that NSAIDs raise the mean blood pressure by 5 mmHg [3], and attenuate the effects of anti-hypertensive medications such as angiotensin-converting enzyme (ACE) inhibitors, beta-blockers, and diuretics [4]. Different NSAIDs vary significantly in their effect on blood pressure with indomethacin, naproxen, and piroxicam resulting in the highest elevations in blood pressure [5]. Modest elevations in blood pressure are usually seen, with significant rises causing hypertensive crises rarely being reported in the literature [6]. 

Due to the lack of reports on this topic, and to highlight the effect of NSAIDs on blood pressure, we present a case of a 58-year-old female who presented with a hypertensive crisis shortly after consuming indomethacin for her frozen shoulder.

## Case presentation

 A 52-year-old female was prescribed indomethacin, 75 mg extended-release tablet for frozen shoulder. After she took the tablet of indomethacin, she developed severe blurring of vision around 1 hour later. She also complained of giddiness and had difficulty in writing as she developed tremor in hands. Her blood pressure (BP) measured immediately was 210/170 millimeter of mercury (mmHg) and the remaining vitals were within the normal range. The baseline BP of our patient was 100-110/60-70 mmHg. 

She had a history of migraine and was taking metoprolol for prophylaxis. She also had a past history of hepatitis B infection 33 years back. She has a family history of hypertension and diabetes mellitus in her father. 

She was immediately rushed for an MRI and developed a throbbing headache on the way. Her BP was measured to be 180/110 mmHg and the measurement 2 hours later was 140/90 mmHg. Similarly, BP measurement in the evening was 120/80 mmHg. She remained asymptomatic on the next day and her BP that day was 120/70 mmHg. She had not been prescribed NSAIDs or any other medications. However, she again developed a severe throbbing headache with an elevated blood pressure of 190/110 mmHg on the third day.

**Figure 1:**
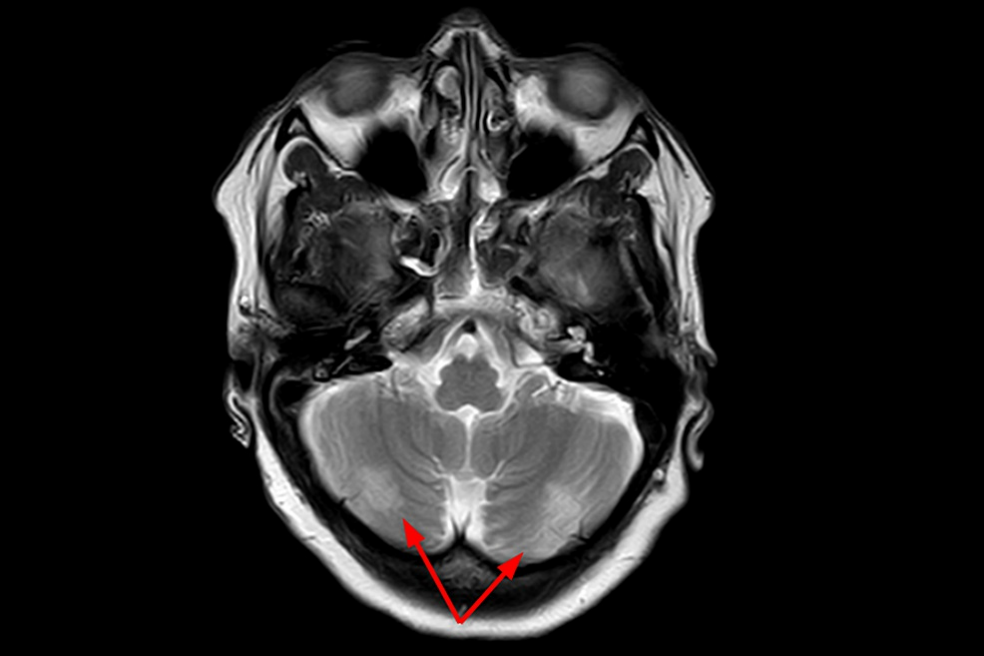
T2-weighted MRI of the brain-axial section showing subtle focal patchy hyperintensities in bilateral cerebellar hemispheres (arrows)

She was monitored clinically and laboratory investigations were not performed. A CT scan of the head was done to rule out intracranial bleeding and it came out to be normal. On MRI of the head, T2-weighted images revealed subtle ill-marginated patchy high-signal-intensity areas in bilateral cerebellar hemisphere in posterior aspect involving cortical area (Figure 1). The patient was not admitted; rather home management was done as her tremor, giddiness, and headache subsided quickly. Similarly, her BP readings returned to baseline on the fourth day of symptoms' onset and afterward. 

 

## Discussion

It is estimated that more than 29 million adults consume NSAIDs regularly and that 73 million prescriptions of NSAIDs are written every year [1,7]. In a span of five years, from 2005 to 2010, there was almost 41% increase in the NSAID use [1]. Although NSAIDs are most commonly used for their anti-inflammatory, antipyretic, and analgesic properties, they have shown to offer protection against cancer and coronary artery disease as well [1]. Since NSAIDs are the most common options available for the treatment of mild and moderate pain, and given the proven efficacy of NSAIDs in widespread clinical disorders, the use of NSAIDs seems to be unavoidable [1,8,9]. As the use of NSAIDs is rising rapidly, the adverse effects are becoming more concerning. Even though gastrointestinal toxicity is the major limiting factor in the use of NSAIDs, various organs of the body can be involved [8]. The effect on each organ system varies significantly among NSAIDs. We presented a case of a 57-year-old female who developed cardiovascular and neurological manifestations after use of indomethacin, a commonly used NSAID. 

Although indomethacin increases blood pressure in certain patients, those with pre-existing hypertension are at greatest risk [5,10]. Indomethacin increases blood pressure in patients controlled on ACE inhibitors, but minimal effect is seen on patients under calcium channel blockers. Adequate data regarding effects of NSAIDs on patients under diuretics or beta blockers are lacking [4]. NSAIDS inhibit cyclooxygenase 2 in the kidneys, which is associated with reduced prostaglandin I2 and this may be responsible for their hypertensive action. Reduced prostaglandin I2 is responsible for reduced sodium excretion and increased intravascular volume [11]. It is not clear whether different NSAIDs have a variable effect on the blood pressure but what is seen is that indomethacin, naproxen, and piroxicam are associated with the clinically significant changes in the blood pressure among all the NSAIDs [12]. The average rise in blood pressure is 3 mmHg systolic and 2 mmHg diastolic, but in our case patient's baseline blood pressure was 100-110 mmHg systolic and 60-70 mmHg diastolic, which was raised to 220/110 mmHg [13-15]. 

Aseptic meningitis, psychosis, and cognitive dysfunction are the commonly reported neurologic side effects of NSAIDs [16,17]. Our patient had developed headache, giddiness, and tremor, which are the uncommon neurologic manifestations of NSAIDs. The symptoms our patient presented were partly explained by cerebellitis seen in MRI. An attack of migraine along with the elevated blood pressure could have caused these manifestations. Although minor changes in blood pressure are frequently seen with NSAID use, the manifestations shown by our patient are uncommon in the literature. 

## Conclusions

NSAIDs are one of the most commonly used medications and the adverse effects of NSAIDs are raising concerns with the increasing use of these drugs and effect on blood pressure is one among them. The effect of NSAIDs on blood pressure is varied with a modest increase seen with most drugs and the effect is seen primarily in hypertensive patients. In our case, a normotensive patient presented with hypertensive crisis and neurologic manifestations like tremor, both of which are uncommon in the literature following the use of indomethacin, an NSAID.
